# Staining Artificial Teeth to Match Exceptional Natural Teeth

**Published:** 1884-03

**Authors:** William Dunn

**Affiliations:** Florence


ARTICLE VI.

### STAINING ARTIFICIAL TEETH TO MATCH EXCEPTIONAL NATURAL ONES.

BY MR. WM. DUNN, JUN., OF FLORENCE.

As in the *British Journal of Dental Science*, for December last, it is stated that it would be a great desideratum to be able to stain or color artificial teeth as to make them exactly represent any exceptional natural ones that they may be required to match, it may interest your readers to know that the means are at command which will enable them to exercise their utmost ingenuity and artistic taste to this end, and to accomplish their wishes with really very little trouble to themselves and to the greatest appreciation of their patients. The process which I have adopted for some time in actual practice is as follows ;

Thanks to the exertions of Mr. Lacroix, a chemist, in Paris, we have every requisite color ready to our hands prepared for porcelain painting and put up in color tubes similar to all moist artists' colors, which can be obtained of Lechertier Barbe & Co., 60 Regent's Street, London. The colors which I generally use are : yellow, green, dark blue, brown, pink and black ; all these colors are readily dissolved by essence of turpentine or essence of mint, when with a fine camel hair pencil they may be blended in any combinations, and the surfaces, necks, tips or other parts painted, tinted, lined or otherwise made to perfectly coincide with any natural teeth however irregular or particolored they may be. Of course in most instances it is necessary to grind or disfigure the artificial teeth before painting them, and thus may be represented the worn down teeth of advanced age which so frequently show the dark brown tinted dentine within a circle of paler enamel, or the dark ring of enamel with the lighter centre; the stained crowns of the bicuspids and molars under similar circumstances, the dark necks of denuded teeth, although the enamel surface may remain a good color, the blue-black appearance of carious teeth, either stained naturally or from amalgam stoppings. If naturally, it is very effective to grind notches out of the artificial teeth to represent decay and stain within and around the parts so disfigured. Honey-combed teeth may also be well represented by similar treatment. Indeed, there is practically *no limit* to the capabilities of the materials in skillful and artistic hands.

I find a *white* pallet advisable for mixing the colors.

After the teeth are painted they should be placed on pieces of platina or platina gauze, or in separate crucibles, for care should be taken that they do not touch one another.

If a whole set has to be colored it will be found convenient to fix them in a row in a mixture of two parts of plaster of Paris and one part of fireclay—which will not crack in firing. In this way a more uniform tint can be given than if done separately.

For firing I prefer the handy and simple but effective little gas furnace invented by Mr. Verrier, in which it takes about five minutes. The sufficiency of heat can easily be determined by watching until the crucible becomes a cherry red. Care must be taken not to overheat otherwise the colors—especially the lighter ones, will disappear. These colors are as a general rule unalterable, but the operator must bear in mind that in passing through the vulcanizing process they are apt to become a little darker, and regulate his tints accordingly. At the same time the darkness can be corrected, if necessary, by applying fine sandpaper or pumice powder.

With the pink color a very fair imitation of the gums can be produced; quite efficient in most cases of a single tooth, to prevent that disagreeable and unsightly appearance so often seen, of a long tusk, rather than tooth, between two short teeth.

With a little attention and practice anyone may soon master the initial difficulties of this painting work, and few, once having seen the advantages of the results, will again be content with the old method of using the nearest color attainable.—*British Journal of Dental Science.*

